# Collaborative Research and Development of a Novel, Patient-Centered Digital Platform (MyEyeSite) for Rare Inherited Retinal Disease Data: Acceptability and Feasibility Study

**DOI:** 10.2196/21341

**Published:** 2022-01-31

**Authors:** Rose M Gilbert, Dayyanah Sumodhee, Nikolas Pontikos, Catherine Hollyhead, Angus Patrick, Samuel Scarles, Sabrina Van Der Smissen, Rodrigo M Young, Nick Nettleton, Andrew R Webster, Jocelyn Cammack

**Affiliations:** 1 NIHR Moorfields Biomedical Research Centre Moorfields Eye Hospital NHS Foundation Trust and UCL Institute of Ophthalmology London United Kingdom; 2 Medical Retina Service (City Road) Moorfields Eye Hospital NHS Foundation Trust London United Kingdom; 3 Addenbrookes Hospital Cambridge University Hospitals NHS Foundation Trust Cambridge United Kingdom; 4 Florence Nightingale Faculty of Nursing, Midwifery and Palliative Care Kings College London London United Kingdom; 5 Institute of Ophthalmology UCL London United Kingdom; 6 MyEyeSite IRD Patient Advisory Group London United Kingdom; 7 Loft Digital Limited London United Kingdom

**Keywords:** MyEyeSite, inherited retinal diseases (IRD), rare diseases, genetics, ophthalmology, digital health, eye data, GDPR, subject access request (SAR), mobile phone

## Abstract

**Background:**

Inherited retinal diseases (IRDs) are a leading cause of blindness in children and working age adults in the United Kingdom and other countries, with an appreciable socioeconomic impact. However, by definition, IRD data are individually rare, and as a result, this patient group has been underserved by research. Researchers need larger amounts of these rare data to make progress in this field, for example, through the development of gene therapies. The challenge has been how to find and make these data available to researchers in the most productive way. MyEyeSite is a research collaboration aiming to design and develop a digital platform (the *MyEyeSite platform*) for people with rare IRDs that will enable patients, doctors, and researchers to aggregate and share specialist eye health data. A crucial component of this platform is the *MyEyeSite patient application,* which will provide the means for patients with IRD to interact with the system and, in particular, to collate, manage, and share their personal specialist IRD data both for research and their own health care.

**Objective:**

This study aims to test the acceptability and feasibility of the MyEyeSite platform in the target IRD population through a collaborative patient-centered study.

**Methods:**

Qualitative data were generated through focus groups and workshops, and quantitative data were obtained through a survey of patients with IRD. Participants were recruited through clinics at Moorfields Eye Hospital National Health Service (NHS) Foundation Trust and the National Institute for Health Research (NIHR) Moorfields Biomedical Research Centre through their patient and public involvement databases.

**Results:**

Our IRD focus group sample (n=50) highlighted the following themes: *frustration with the current system* regarding data sharing within the United Kingdom’s NHS; positive *expectations* of the potential benefits of the MyEyeSite patient application, resulting from increased access to this specialized data; and concerns regarding data security, including potentially unethical use of the data outside the NHS. Of the surveyed 80 participants, 68 (85%) were motivated to have a more active role in their eye care and share their data for research purposes using a secure technology, such as a web application or mobile app.

**Conclusions:**

This study demonstrates that patients with IRD are highly motivated to be actively involved in managing their own data for research and their own eye care. It demonstrates the feasibility of involving patients with IRD in the detailed design of the MyEyeSite platform exemplar, with input from the patient with IRD workshops playing a key role in determining both the functionality and accessibility of the designs and prototypes. The development of a user-centered technological solution to the problem of rare health data has the potential to benefit not only the patient with IRD community but also others with rare diseases.

## Introduction

### Inherited Retinal Diseases and Challenges in Accessing Eye Data

Inherited orphan rare eye diseases are genetic eye diseases that have not been adopted for drug development. This group of conditions includes inherited retinal diseases (IRDs), an umbrella term for lifelong genetic conditions that affect the retina. Although *rare diseases* may be individually rare, they are collectively common, with 1 in 17 people being affected by a rare disease at some point in their lives [1]. IRDs are a leading cause of blindness in children and working age adults in the United Kingdom and other countries, with an appreciable socioeconomic impact [2,3]. People with IRD face a range of challenges that are both directly and indirectly related to their eye condition. These may include a delayed or inconclusive diagnosis (which can be especially challenging for children with IRD and their caregivers) and no access to curative treatment, difficulty accessing specialist clinicians with expertise in their condition, a lack of awareness from others regarding their eye condition, and a myriad of other disease-specific quality of life issues [4,5].

Eye care for IRD is highly specialized, requiring complex genetic information as well as large amounts of clinical data, including high-dimensional ophthalmic images, such as optical coherence tomography scans, taken over the course of a patient’s lifetime [6,7]. Therefore, although rare eye diseases are often analyzed at a great level of detail (deeply phenotyped), these specialized data tend to be *siloed* and fragmented across multiple specialist sites, making it difficult for doctors (most often ophthalmologists) to access all relevant data regarding a patient before an appointment. Doctors are often reliant on patients to collate their data from various sources and bring it with them to an appointment. In the United Kingdom, patients are not given easy access to their medical records, which has led to a culture of private digital data *hacks,* including taking photos of medical letters and computer screen displays and bringing these to a medical appointment. We do not believe that this is an acceptable situation as it introduces risks with regard to patient data confidentiality and information governance (IG), which could be mitigated by providing more robust and efficient systems for sharing patients’ health data. Furthermore, it runs contrary to the principles of the UK General Data Protection Regulations, which state that data should be made available in an “accessible, concise and intelligible format” and “disclosed securely” [8].

Difficulty accessing IRD data is further compounded by the lack of publicly available natural history information for specific gene mutations relevant to IRD and poor knowledge regarding the correlation between clinical observations (objective measures) and first-hand experiences of IRD reported by the affected people (subjective measures) [9]. The incidence of the early stage disease remaining undetected or being misdiagnosed [10] because of lack of data makes it more difficult to ascertain the prevalence of these diseases, especially at a genetic level. Streamlining the process of secure health data sharing at both national and international levels while delivering data with the required level of clinical and technical details for clinical and research purposes is a challenge. However, it has the potential to transform health outcomes [11] and positively affect the cost of IRD both for individuals and health services [2]. It is also likely to accelerate the process of therapeutics discovery, including gene therapies [12,13], through enhanced recruitment in clinical trials, an ambition that aligns with the United Kingdom national health research strategy to create the best research for the best health [14].

As IRDs are individually rare, there is a lack of disease-specific data for every IRD, which is suitable for research. The available data are widely dispersed and difficult to consolidate. This is a limiting factor in research, and as a result, the patient with IRD population is underserved compared with patients with more common disorders. To address these issues, researchers need access to comprehensive, linked, longitudinal phenotypic, and genomic data in a suitable format. The data need to be extracted from patient registries across the United Kingdom National Health Service (NHS) hospital systems and other sources, linked to genomic data and prepared (coded) for research. Patient registries are an essential tool for increasing current knowledge regarding rare diseases [15,16]; however, the way the registry is designed and organized is key. Attention must be paid to making the data findable, accessible, interoperable, and reusable and to IG and data quality, among other aspects, of the database architecture [17].

**Figure 1:**
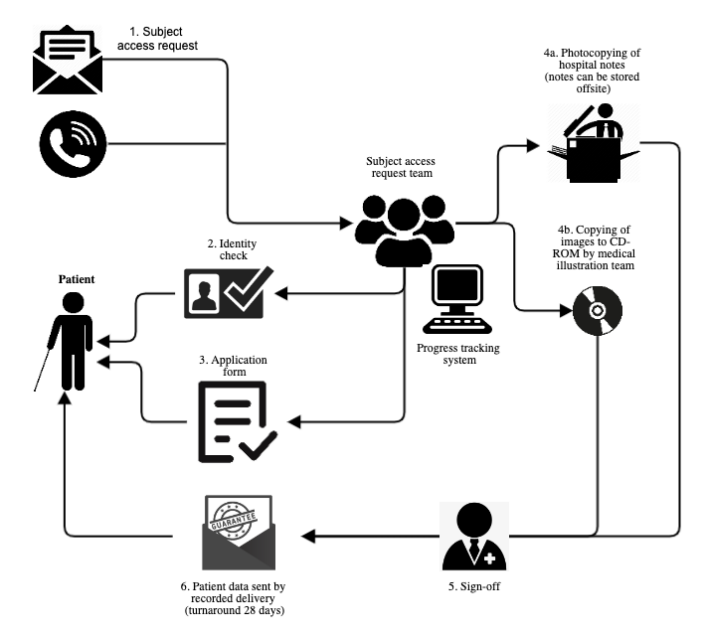
Example of subject access request process at Moorfields Eye Hospital National Health Service Trust.

Patients have the right to access their data, as laid out by the General Data Protection Regulations [8,18,19]. Obtaining personal copies of detailed medical data requires the patient to submit a subject access request (SAR) to an individual NHS Hospital Trust, a procedure that requires a response within 4 weeks. The data returned to the patient is often in the form of photocopied paper notes and a CD of images, if available (Figure 1). Although the SAR process is unsustainable at scale, it stands to reason that the person best placed to initially access a complete individual data set is the patient with IRD. The experiences of living and dealing with a rare disease [20] make patients with IRD a particularly motivated population to catalyze digital innovation for patient benefit. Therefore, we propose an IG-compliant platform and application (MyEyeSite) for digitally processing, sharing, and storing eye data at the necessary scale and speed, which is managed by patients with IRD without the need for special expertise.

### The MyEyeSite Project: Aims and Approach

The MyEyeSite project is a collaboration among Moorfields Eye Hospital NHS Trust, Loft Digital, and University College London Institute of Ophthalmology, which began work on a feasibility study funded by the Health Data Research United Kingdom in 2019. This project adopted user-centered design principles and a particularly patient-centered approach as fundamental to its methodology. In striving to address the myriad of challenges of data linkage across the NHS outlined above (detailed in Multimedia Appendix 1 [2,21-27]), the project set out to establish digital tools and streamlined workflows to help individuals in the IRD community access and make the best possible use of their eye health data through 5 specific aims (Textbox 1).

**Aims of the MyEyeSite platform and patient application.**

**Aims of the MyEyeSite platform and patient application**

- To aggregate consented rare disease data into a centralized electronic location
- To exploit the unique position of the patient as pivotal in this process through the subject access request system and ensure their access to their own data for self-management of their care (this also meant ensuring that the MyEyeSite patient application was fully accessible to those with severe sight impairment)
- To enable access to the data for clinicians and researchers in a safe and compliant manner so as to increase research on inherited retinal diseases
- To facilitate the communication of data and, therefore, knowledge between secondary and tertiary treatment centers to enhance treatment
- To develop a model of data curation that could be an exemplar for rare diseases in other medical specialties

**Figure 2:**
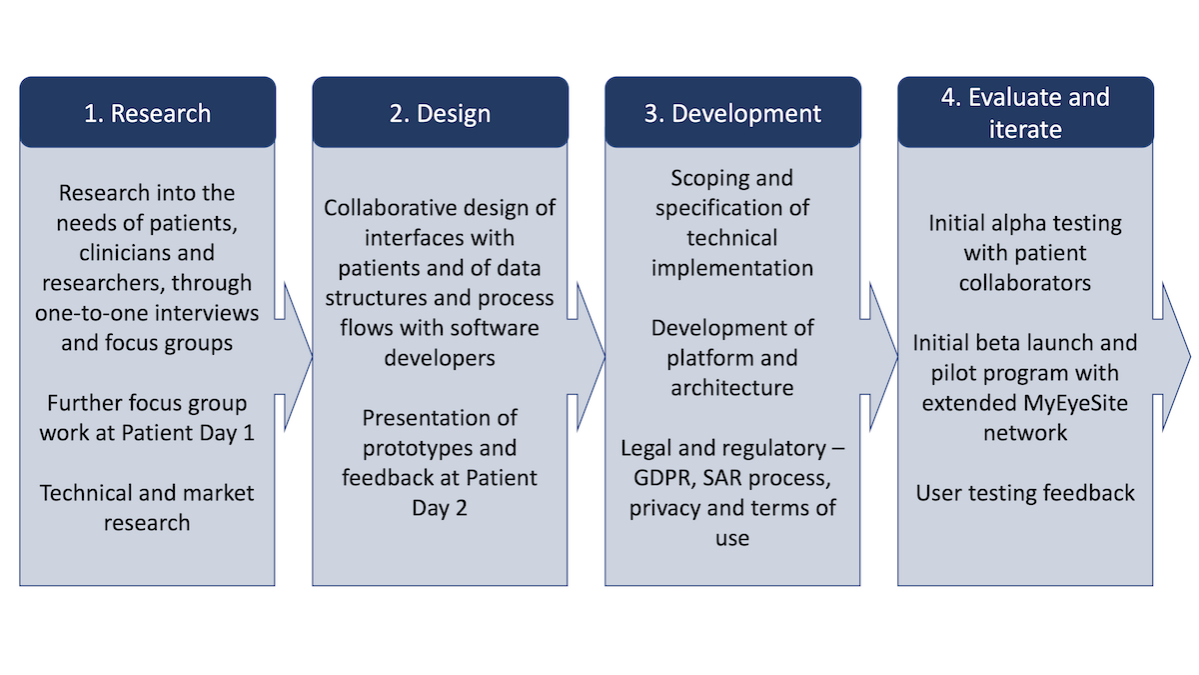
High-level schematic of the work program to develop the MyEyeSite platform. GDPR: General Data Protection Regulation; SAR: subject access request.

The scope of the initial program of work was to evaluate the feasibility of the endeavor from a range of perspectives (involving user, market, technical, and security research) and to develop a prototype of the proposed platform and patient application that could be tested on smartphones, tablets, or desktop computers. The process for this phase of development is outlined in Figure 2.

**Figure 3:**
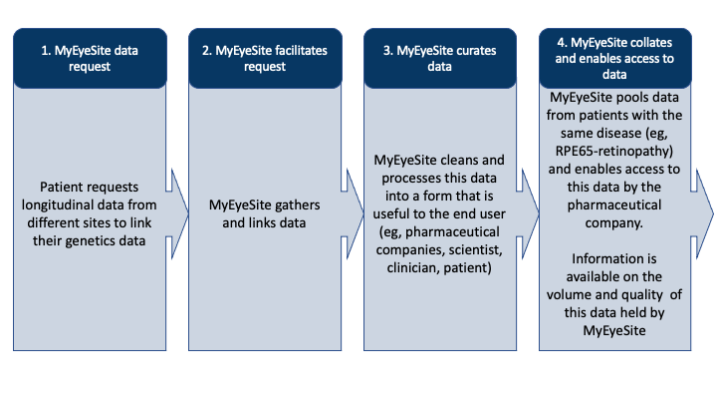
MyEyeSite: patient data request process.

**Figure 4:**
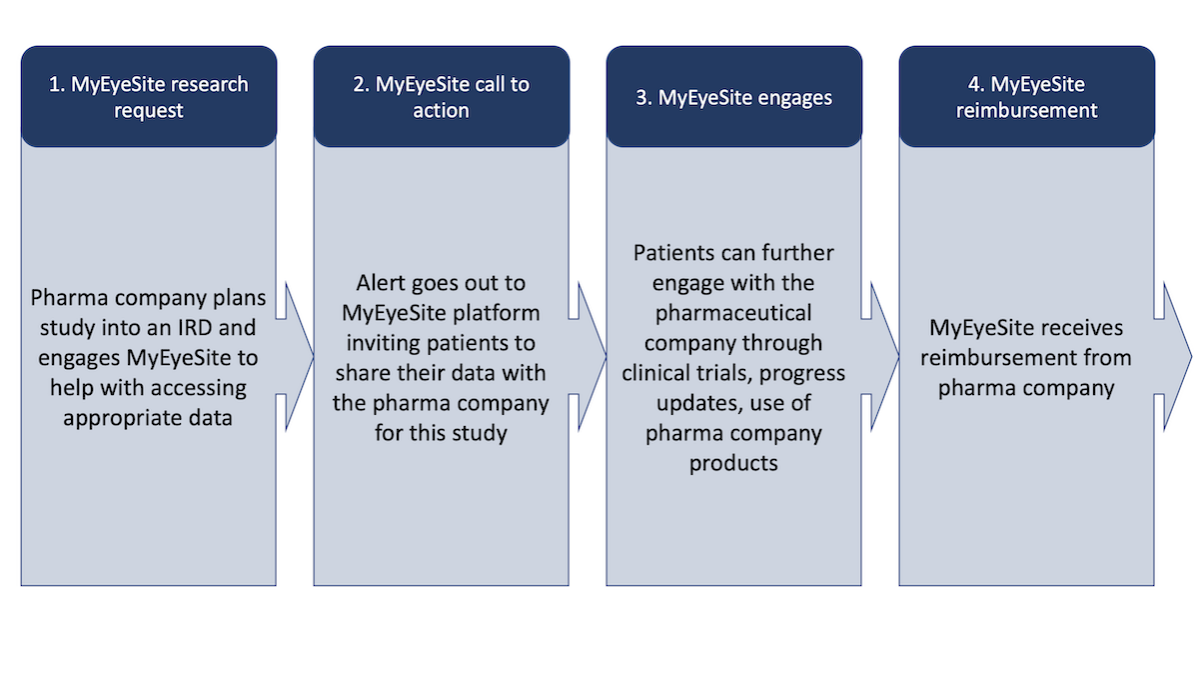
MyEyeSite: researcher data request and engagement process. The request example used here is from a pharmaceutical company. IRD: inherited retinal disease.

An enriched picture of future objectives and uses for the technology was also established based on use-case scenarios (Textbox 2; Figures 3 and 4).

The MyEyeSite *use case* is outlined in Figures 3 and 4.

**Intended uses of the MyEyeSite platform.**

**Intended uses of the MyEyeSite platform**

- To facilitate subject access requests from patients to hospitals for disease-appropriate data
- To provide a framework for hospitals to respond efficiently to such patient requests
- To allow patients to access their own data in an informative way, robust to sight impairment
- To provide pooled data on consented patients for research purposes

The underlying strategy of the MyEyeSite platform is to connect groups of patients with IRD with similar conditions and uniquely valuable data sets to research projects and clinical trials with the potential to restore sight using a patient-centered methodology.

### The MyEyeSite Project: Patient Engagement Study Design and Objectives

There is a high expectation among patients regarding the quality of communication between health care providers and themselves [28,29], and this is consistent with the shift in medicine toward patient-centered care. Thus, it has become the accepted best practice to engage patients in the design process of eye care interventions [30-35]. A study involving patients with retinitis pigmentosa, a relatively common phenotype within IRD, showed that engagement strategies significantly affected the vision-related quality of life and emotional health, with disengagement having a particularly negative effect [36]. Furthermore, the UK Medical Research Council guidance on developing complex interventions states that the best practice is “to develop interventions systematically, using the best available evidence and appropriate theory, then to test them using a carefully phased approach, starting with a series of pilot studies targeted at each of the key uncertainties in the design, and moving on to an exploratory and then a definitive evaluation” [37].

The first phase of developing the MyEyeSite platform intervention focused on engagement with our target population: the patient with IRD community. We aimed to follow the principles set out in the UK government’s Department of Health and Social Care’s policy paper [38], which describes the involvement of users throughout the research, design, and build process.

The objective of this study was to test the acceptability and feasibility of the MyEyeSite platform. For the purposes of developing the MyEyeSite platform, stakeholders were engaged via multiple methods, which included individual interviews, focus groups, a web-based survey, professional networks, conference presentations, and public engagement events. Participants provided verbal and written consent for their data to be used for this project. According to the UK NHS Health Research Authority and Medical Research Council guidelines on defining research [39], this pilot study was not deemed a formal research study, and so, NHS ethical approval was not required.

## Methods

### Thematic Analysis of Focus Groups and Workshops

#### Recruitment

Patients with IRD were specifically recruited for MyEyeSite focus groups, technology design, and user research workshops via specialist retinal clinics at the Moorfields Eye Hospital in London and across the United Kingdom, patient charities (The Macular Society, Retina United Kingdom, Bardet–Biedl Syndrome United Kingdom, and Fight for Sight), and a patient registry held by the National Institute for Health Research (NIHR) Moorfields Biomedical Research Centre.

#### Patient and Public Involvement and Thematic Analysis

Focus groups were conducted with 5 participants in each group in June 2019 at a patient and public involvement event in London. Following the interviews, a sample size of 20 was finalized when no new codes or themes were identified, and it was concluded that saturation had been reached. A total of 2 technology design workshops were held in November 2019 and attended by a further 30 participants to discuss the MyEyeSite patient application design prototype, which had been prepared in Adobe XD. The application interface was presented to the participants on a large screen that they were able to access either directly or by taking a photo of the screen on their mobile device and magnifying that image to suit their personal needs. Screen-reader technology was also used in these workshops.

The 50 participants ranged in age from 10 to 75 years. The group was informed of the purpose of the discussions, how the data would be used, and how to withdraw from the group. Informed consent was obtained for participation and use of their data to develop MyEyeSite. The discussions followed a semistructured guide exploring how the patients would feel regarding their clinical data being stored on a digital platform and subsequently being used for research. Patients’ experiences and concerns regarding sharing data with universities and pharmaceutical companies were also investigated. The focus groups (moderated by RMG, ARW, NN, SS, and SVDS) averaged 58 (SD 1.2) minutes and were audio taped and transcribed verbatim, with participants deidentified using pseudonyms. A short film demonstrating some of these approaches is available to view as a QuickTime (Apple, Inc) video file in Multimedia Appendix 2.

An inductive thematic analysis was conducted to analyze the data following the Braun and Clarke 5-step guide [40,41]. This method was chosen to analyze the qualitative (transcribed) data for the following reasons: it is a useful method for working within the participatory research paradigm (with participants as collaborators), it can usefully summarize the key features of a large body of data, it can generate unanticipated insights and allow for social as well as psychological interpretations of data, and its results are generally accessible to the educated general public.

**Figure 5:**
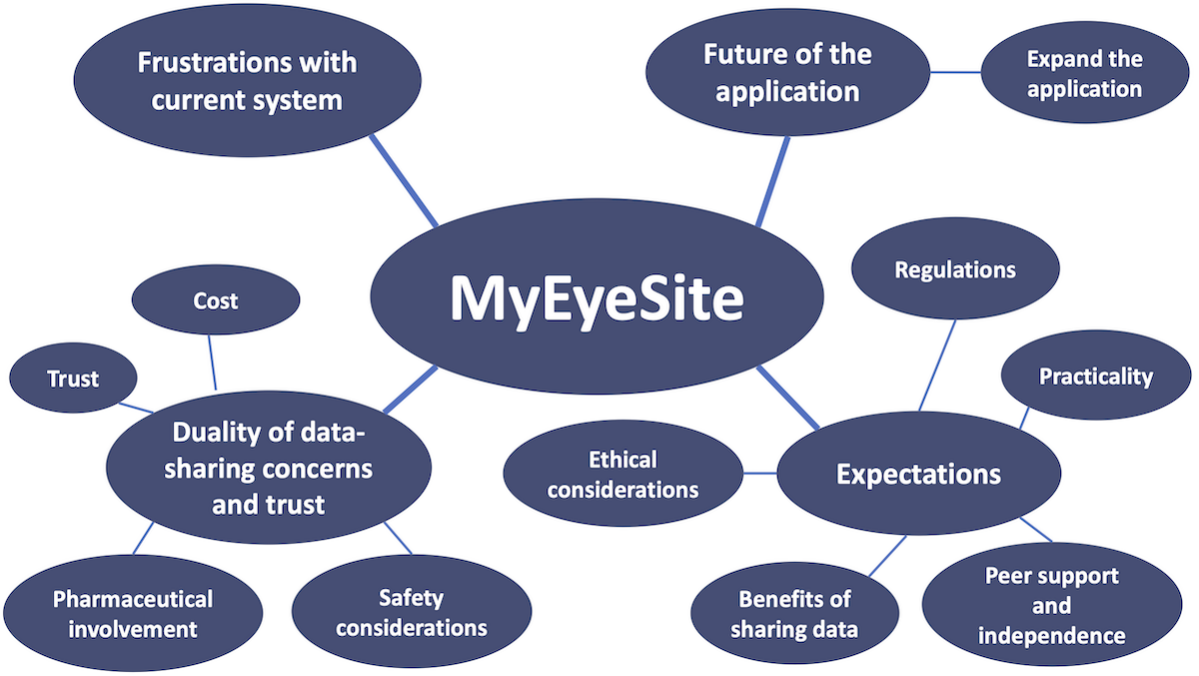
Thematic map depicting analysis of the focus group and workshop discussions.

The transcripts were read and reread to understand the depth of the data and then transferred into NVivo (version 12 QSR International), a computer-assisted qualitative data analysis software tool for organizing, storing, and analyzing transcribed data [42], where the codes were initiated. Following this, the codes were combined into themes. The creation and discussion of the themes took place during face-to-face meetings between the authors to make sure that those themes were applicable to the codes, true to the data set, and reflective of the meaning that the participants intended. Finally, the themes were defined, transcript quotations were chosen to illustrate the themes, and a *thematic map* was produced (Figure 5).

### Web-Based Survey of Patients with IRD

We conducted an anonymous Google Forms survey (Multimedia Appendix 3) of views within the IRD community regarding the sharing of eye data, which was circulated via patient charities, eye clinics, social media, and our project’s website.

## Results

### Qualitative Results

The thematic analysis of the focus group and workshop discussions outlined four main themes (Figure 5): (1) frustration with the current system, (2) expectations from a digital application, (3) duality of concerns and trust, and (4) future of the MyEyeSite platform.

#### Theme 1: Frustrations With the Current System

The IRD focus group participants expressed frustrations with the current NHS eye data system, which stores their data locally in each hospital and does not allow these data to be easily accessed and shared across hospitals. Patients highlighted the need to have easy access to their data so they could share it with other health care providers involved in their care. Some reported that they kept all of their paper hospital records with themselves so that they could bring them to every eye clinic appointment. They expressed interest in having all their digital data in one electronic location, which they could access through an application such as the MyEyeSite patient application. The following quotation from a patient with IRD highlights an instance where she relocated to a different geographic area, which resulted in the loss of all her eye data, including her family history map of IRD:

From a personal perspective as well, we wouldn’t have lost our data and my mother would be able to know which side of the family all this came down from. So, if I’d have had that data we would still have that along with my letters from 1995. Yeah so, you will not lose your own data because it’s so important to you whereas we are just 1 in 60 million people in the United Kingdom.

Participant 6

#### Theme 2: Expectations of the MyEyeSite Patient Application

##### Benefits of Sharing Data, Peer Support, and Independence

Participants felt that there would be real benefits to using a patient-focused application for tackling the current issues of disaggregated NHS data, which would obviate the need to make individual, institutional data requests via a SAR process. They felt that an application might allow them to participate in research trials with potentially positive health outcomes as a result of accelerated research processes. Participants also described a sense of increased agency and being empowered to manage their conditions through a central place where they could self-administer changes in their personal details and be in control of their own health data, including how it was used in research:

But if they got in touch with that particular patient and explained the reason why they want to copy it, and then we can give that permission, so it’s granted by the patient.

Participant 4

The desire for an application to facilitate a sense of community through the linkage of patients with similar eye conditions was strongly expressed:

I mean there’s a great need for people to know that there are other people around that have the same problem or a similar problem. There’s great need.

Participant 5

I was going to say...is there any value in links maybe? If you suddenly feel totally isolated and you’ve just been told that you’ll lose your eyes and nothing can be done, however these links may be of use to you in your area.

Participant 3

##### Practicality of the Application: Accessibility

**Figure 6:**
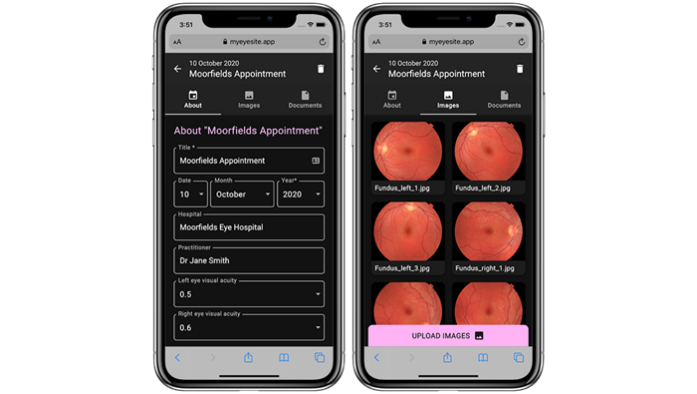
Example of the MyEyeSite patient application front-end design for a smartphone.

The user research workshop sessions, which tested the *front-end* design of the patient application (Figure 6; Multimedia Appendices 4 and 5), highlighted diverse accessibility requirements, even within the patient with IRD group.

Patients had different requirements of the visual display according to their diagnosis, level of disease progression, and disability from vision loss. Detailed discussions led to an expansion of the available accessibility features so that, in addition to user-friendly fonts and common color options (normal, high contrast, and dark mode), settings were introduced for users who have difficulty perceiving specific colors. Some patients relied on a screen-reader software such as Job Access With Speech (Freedom Scientific, Inc) [43] to access information. It was also apparent that some older adults found it difficult to access or navigate the applications because of a lack of experience with digital tools, as well as vision problems. These individuals often relied on their neighbors, friends, or families for assistance.

Participants felt that any application should be accessible both on their smartphones and via a desktop (using a web browser) and expressed the need to have their data available in lay terms that avoid medical jargon. User-friendly access to the application could also allow them to perform some simple analyses and plot graphs to self-monitor their condition.

##### Practicality of the Application: Functionality

Sharing of data in an anonymized form was perceived as a necessary safety feature by participants:

I would be happy to share my clinical information if it helps research or...but then maybe to disconnect my personal sort of data away from that.

Participant 3

If people want to access that anonymous data that’s got genetic information and my age or whatever it might be, but nothing else that identifies me, you don’t have to ask me every time for that.

Participant 6

Participants expressed a desire to have an easy *opt-in* and *opt-out* option to take part in research studies and receive notifications to let them know whether they were eligible to participate and whether there were any significant findings from the research in which they participated:

The other bit for me was the research piece, which was to say anonymised data; people can access my clinical data [but] I would want to make sure individual [research] requests come through to me.

Participant 6

##### Regulations and Ethical Considerations

Participants suggested a form of recognition or incentives for sharing their data with parties external to the NHS and called attention to the need for clear policies, strict controls, and security systems in any digital technology to protect their personal details from people who might use it for unethical purposes. In particular, they asked for transparency and traceability regarding access to their data, as highlighted by the following quotations:

So, knowing, I suppose, where that data’s going, who’s got the data, or what it could be used for, the potential research...just knowing kind of an overview of what it’s for, what the reason this is for, then people can make that decision. I think it’s important.

Participant 1

If, for example, I was contacted by say an outside agency, an organisation, whether it was a researcher or if I was being, you know, [added] into some clinical trial and the clinical team wanted access to that, I would want to be asked, I would want to know exactly what that meant before I gave my consent.

Participant 3

Participants raised further ethical concerns regarding decision-making around data sharing outside the NHS and power of attorney agreements with regard to data sharing for minors or severely sight-impaired patients:

You take away the competition don’t you if you give them [pharma company] exclusivity. Then they can take their time and they might [take] another ten years over it.

Participant 7

If somebody’s got power of attorney already, does that mean automatically they now get access to this sort of thing? Because power of attorney means I can do what I need to do for you financially, but does that mean they can also access this or because I’ve gone doolally or whatever it is, I might have said, okay go do what you need to do - is this separately controlled or is it covered by that piece of the legality?

Participant 8

#### Theme 3: Duality of Concerns Around Data Sharing and Trust

Trust was identified as a key requirement for any data sharing agreement. Participants were motivated to share their data with clinicians, as they felt that this would contribute to finding a cure for their conditions:

I’m happy for the doctors, the opticians, the clinical side of people that I see, I would want them to have access to my information. That’s absolutely fine. And it’s for the right reasons. They’re looking for a cure so it’s all good isn’t it?

Participant 3

On the other hand, participants acknowledged the need for a balance between data accessibility and security:

So, for me the app, I like the idea of it. I like the idea of the bigger picture, which is, you know, what [we’re] talking about, that kind of having that data that can help future research. I really like that sort of idea, but it’s the security of it...it’s just like if you lose your phone or—could this be manipulated in some way? It could be misused in some way.

Participant 5

As patients we would want to make sure the app is secure. We would want a secure platform and some assurances behind that. I think the ability that the patient controls the access to whoever and is reminded on a regular basis you’ve given that access to people, to review it.

Participant 11

I want to be able to see a log of who has accessed it and when.

Participant 9

I think giving somebody a period of access with that: okay I’m happy to let that person be able to see it for three months, I’m happy to let that person see it for six months. Or, every year they come back and go, did you remind yourself that you actually had given permission to these people to access your data? Do you want to turn it on or off for anyone?

Participant 6

To address some of these concerns, an external organization was invited to perform penetration testing on the application, and the messaging system between clinicians and patients was also improved to keep the patients better informed and help them feel more secure.

#### Theme 4: Future of the MyEyeSite Platform

Some participants felt that if a platform for IRD data were developed, then as many patients as possible should be encouraged to use it to gain the most benefit for the community. Hence, they would support international data sharing, as it would allow the creation of larger data sets:

And to get to individualised care we have to have sharing of data in big groups of people because these diseases are all rare. I mean, achromatopsia there’s only 35 people in the UK. So, you know, that needs to be international, I think.

Participant 1

### MyEyeSite Survey Analysis and Quantitative Results

#### Demographic and Diagnostic Survey Data

The MyEyeSite survey was completed by 80 participants between April 2019 and June 2020. Of these 80 participants, 71 (89%) identified as White, 9 (11%) as Asian or Asian British, and 1 (1%) as *other.* Of the 80 respondents, 40 (50%) identified as female, and 66 (83%) were equally distributed across the ages of 25 to 65 years. The highest levels of educational attainment among the respondents were as follows: 34% (27/80) had an undergraduate university degree, 25% (20/80) had a nonuniversity college qualification, 23% (18/80) had a postgraduate university degree, 10% (8/80) reported secondary school as their highest educational level, 6% (5/80) had technical or vocational education, and 4% (3/80) had primary school level education.

Of the 80 survey respondents, 98% (n=78) specified their IRD diagnoses as follows: 53% (n=42) had retinitis pigmentosa, 15% (n=12) had Stargardt disease, 25% (n=20) had *other* IRD diagnoses, and 5% (n=4) reported that they were *not sure* of their IRD diagnosis. A breakdown of the 25% (n=20) of patients reported *other* IRD diagnoses.

With regard to the prevalence of additional eye conditions to IRD, cataract was the most common, affecting 19% (15/80) of participants, followed by 5% (4/80) having age-related macular degeneration and 10% (8/80) of respondents (2 for each condition) reporting glaucoma, retinal detachment, uveitis, and dry eyes. On a scale of 1 to 5, with 1 being fully sighted and 5 being severely sight impaired, 26% (21/80) of respondents self-assessed their level of sight at level 5, 21% (17/80) at level 4, 29% (23/80) at level 3, 10% (8/80) at level 2, and 6% (5/80) at fully sighted (1 person did not respond to this question).

#### Patients’ Current Access to Personal Health Data

Currently, the only route by which patients can access their personal health data from a hospital in the United Kingdom is by making a SAR. Only 18% (14/80) of surveyed respondents had attempted to retrieve their health data this way and 6% (5/80) had not heard of a SAR before. Of those 14 who had performed a SAR, 50% (n=7) had found the process *extremely difficult* (level 5 on a Likert scale of 1-5) or very difficult (level 4), and for 8 (57%) respondents, the process was either wholly or partly unsuccessful in achieving their intended purpose.

The reasons for the completion of the SAR have been listed in Textbox 3. All patients who had independently made a SAR self-assessed their vision at level 4 or 5 on the 1 to 5 scale of vision, with 1 being fully sighted and 5 being severely sight impaired. The MyEyeSite platform is able to accommodate all the reasons they gave for making a SAR, as well as those of nonpatients (Textbox 3).

**Reasons given for making a subject access request for hospital data.**

**Reasons for giving a subject access request**

- To get my son’s genetic testing information (he has Stargardt disease)
- To gather insight for the MyEyeSite project
- For my own curiosity
- To have photos of my retina scans
- For fertility/genetics consulting via Genetics
- French clinical trial
- To support the claim for personal independence payment
- For curiosity and for information when I was pregnant with my son
- I wanted copies of my records to understand what they comprised and understand more about my condition
- Asked to by project team and personal interest
- Hearing test data required by another hospital

#### Patients’ Access to Personal Health Data Using MyEyeSite

Of the 80 surveyed respondents, 85% (n=68) were motivated to have a more active role in their eye care and to share their data for research purposes using a secure technology, such as a web or mobile app. Approximately 14% (11/80) of respondents were unsure whether they would want greater involvement in their own eye care, with 82% (9/11) being >35 years of age. All the survey respondents had access to the internet; however, the 9 respondents were born before 1985 and so would not have grown up with smart technology, a factor that may have had a bearing on their uncertainty regarding data use via smartphone technology. Only 1% (1/80) of respondents wanted no change in the level of their involvement in their own eye care. Of the 80 respondents, 79 (99%) owned a smartphone, and 76 (95%) used apps.

Of the 80 respondents, 44 (55%) had no concerns regarding storing or sharing their health data in a web application or mobile app, whereas 35 (44%) expressed a variety of concerns, as presented in Textbox 4.

**Additional information on respondents’ concerns regarding the use of a health data application.**

**Additional information**

- The effort it might take to get my data from multiple sources into the app
- One of the concerns I have is that this type of information may be shared across social media platforms and could cause upset, misinterpretation, and unnecessary anxiety.
- Third parties getting access such as insurance companies; anything that can hinder my options or impact me financially
- The accuracy of the data and whether it is kept up to date or not (a subject access request is a point in time snapshot for example—as more visits and images happen will this be kept up to date)?
- My concern is that this service would not be available as both a phone app and a regular browser-accessible application for those of us that have great difficulty interacting with smaller devices.
- I would have serious concerns if data were shared with any pharmaceutical companies or equipment manufacturers or suppliers without my consent.
- Genetic information in a phone app
- Many reputable companies have suffered data breaches so you will too.

## Discussion

### Principal Findings

This patient engagement study demonstrates that patients with IRD find it acceptable and highly desirable to be actively involved in managing their own data for increased use in research and in their own eye care. It also demonstrates the feasibility of involving patients with IRD in the detailed design of a technological solution to the problem of paucity in these uniquely valuable data sets. Through qualitative evidence, we identified the specific tradeoffs that patients might find agreeable in practice between the benefits and concerns of eye data sharing. Furthermore, quantitative evidence demonstrated that a substantial proportion (68/80, 85%) of our respondents from the IRD community would like a more active role in their eye care and share their data for research purposes using a secure technology, such as a web application or mobile app. These findings build upon previous studies that suggest that public support for future research uses of data requires greater awareness raising, combined with opportunities for public engagement and deliberation [44]. The findings are also in keeping with a recent large research study of non–eye-specific rare disease patients in Europe [45], which showed that, regardless of the disease severity and sociodemographic profile, patients were supportive of data sharing to foster research and improve health care. However, their willingness to share data came with a specific requirement to respect their privacy, choices, and need for information regarding the use of their data.

This study indicates a clear and unmet need for a collaboratively designed technological solution to clinical data sharing and increased opportunities to participate in health data research within the IRD community. Despite this unmet need, to date, only 18% (14/80) of the study respondents attempted to access their hospital eye health data through the SAR process, and 58% (8/14) of those found this process wholly or partly unsuccessful in achieving their intended purpose.

### Comparison With Prior Work

In 2016, the US-based charity Foundation Fighting Blindness created *My Retina Tracker,* an international web-based registry for people affected with IRD and their unaffected genetic relatives, which allows the sharing of anonymized data with participants, researchers, and clinicians [16]. My Retina Tracker now has 15,700 active patient members, of whom 9% are from the United Kingdom. In the United Kingdom itself, however, there has been no comprehensive shared eye data resource available to the wider community in which the data are adherent to the NHS IG policy and the Information Commissioner’s Office regulations at the point of care in addition to being accessible to eye patients (who may be visually impaired).

As with the My Retina Tracker Registry, the MyEyeSite platform is aimed at accelerating the discovery of therapeutics for IRDs by providing researchers with more efficient identification and selection and enhanced pooling of this rare and hard to find data sourced from around the world. However, the MyEyeSite platform also offers a rare opportunity for patients to *take ownership* of the use of their health data, not only for research but also for their ongoing health care, thereby acknowledging the desire for agency so widely expressed in this study. By moving beyond patients as assets [46], MyEyeSite centralizes the patient as pivotal in gathering together their personal health data from numerous varied databases, in part through the SAR process, with the added benefit of incrementally reducing the need for such individual, institutional data requests and the cost to the NHS of servicing them. My Retina Tracker requires an extensive amount of text data to be manually entered into the web-based registry system, whereas MyEyeSite aims to streamline and simplify this process by automating the SAR process and facilitating the electronic transfer of clinical data, such as optical coherence tomography images, to the platform.

Furthermore, through meaningful collaboration in determining the functionality and accessibility of the technology itself, the user-led methodology by which the MyEyeSite platform and patient applications have been developed has ensured that as many patients’ needs as possible have been addressed while adhering to all due data use compliances. Critically, patients’ ongoing autonomy over consenting to their data being used through the MyEyeSite platform for particular research is transformative when compared with the current model operating within the NHS, which assumes consent for all purposes and removes patient data from all NHS-related research if a patient chooses to opt-out of sharing their data.

The UK Biobank is a large-scale biomedical database and research resource that contains in-depth genetic and health information from half a million UK participants [47]. Although this database is regularly augmented with additional data and is globally accessible to approved researchers, it is not accessible to patients and crucially not linked with their valuable clinical ophthalmic imaging data. MyEyeSite aims to collaborate with the UK Biobank to provide patients access to their stored data and facilitate linkage with their hospital clinical data.

### Limitations

Our survey size of 80 patients was an adequate sample size for this feasibility study, in which we engaged and collaborated with our population of rare eye disease patients. We are currently recruiting a larger sample size for the next phase of the study to implement a prototype of the MyEyeSite platform within our hospital clinical population. Furthermore, we will perform a power calculation to determine the sufficient sample size to conduct a health technology assessment study comparing the novel MyEyeSite intervention with standard care.

Although the MyEyeSite platform will reduce the need for repeated SAR applications and repeated acquisition for consent to share data by giving control of their eye data to the individual patient, each SAR procedure still necessitates meticulous work on the part of the hospital staff in retrieving, deidentifying, and releasing that data.

### Conclusions

The user-led design process used to develop the MyEyeSite platform highlighted the unmet need and strong desire of patients with IRD for enhanced access to their health data and a greater involvement in how these data are used for both health care and research purposes. It also brought to our attention the issue that very few hospitals are equipped to deal with an increasing volume of SAR processes in a timely manner and that evidently, the process does not scale with new or repeated requests.

We conclude that sharing data electronically via a hospital portal or a third-party platform such as MyEyeSite is likely to be the most secure and potentially accessible solution (especially for the visually impaired) to the myriad of challenges of health data sharing at both national and international levels. However, current internal IG regulations in several UK hospitals appear to favor paper correspondence as the mode of communication. Therefore, at the present time, our implementation of the MyEyeSite platform relies on patients uploading and storing their own data and using MyEyeSite as a platform for sharing.

Once available, the MyEyeSite platform will enable patients to gain direct access to their medical records and easily share them for future consultations or second opinions. They will gain insights into their own rare conditions and monitor their progress, treatment, and follow-up. We anticipate that this will improve clinical decision-making and patient outcomes and enhance choice and efficient delivery of health care, even when it is distributed across many health providers and laboratories.

The MyEyeSite platform offers the capacity to connect groups of patients with similar conditions and uniquely valuable data sets to commercial research projects through a system that is managed by the patients themselves. This strategy will facilitate access to a centralized international database of genetic variants, supporting rare disease research and clinical trials that have the potential to restore sight and transform lives. We believe that this model of data curation could be an exemplar for rare diseases in other medical specialties.

## Appendix

Multimedia Appendix 1 Challenges in linking health service data for patient benefit.

Multimedia Appendix 2 QuickTime video of MyEyeSite patient and public involvement activity.

Multimedia Appendix 3 Google Forms survey questions (plain text version) circulated to inherited retinal diseases patients.

Multimedia Appendix 4 MyEyeSite patient app mobile phone user interface showing the Appointments page.

Multimedia Appendix 5 MyEyeSite patient app mobile phone user interface showing retinal images selected for upload to the application.

## Abbreviations

IG: information governance
IRD: inherited retinal disease
NHS: National Health Service
NIHR: National Institute for Health Research
SAR: subject access request
UCL: University College London
