# Supplementary material for: Collaborative Research and Development of a Novel, Patient-Centered Digital Platform (MyEyeSite) for Rare Inherited Retinal Disease Data: Acceptability and Feasibility Study
Source: JMIR Form Res. 2022 Jan 31;6(1):e21341. doi: 10.2196/21341 (PMC8845013; doi:10.2196/21341)
Supplement: Multimedia Appendix 1 [file formative_v6i1e21341_app1.docx]

Multimedia Appendix 1

### Challenges in linking health service data for patient benefit

Linking data across the NHS and internationally would have substantial benefits for patients. Previous studies indicate widespread public support for data sharing and linkage for research purposes and that many believe it would confer benefits such as improved coordination of care, although a range of concerns also exist [1]. In the IRD patient community, there are particular expectations regarding genetic ophthalmic services, which are currently unmet [2, 3], owing in part to the disparate storage of clinical data across NHS care providers.

There are two types of NHS data in the UK: primary and secondary care data. Primary care data, obtained from General Practitioners (GP), which include medical diagnoses and allergies, are electronically stored, which means they can be medically coded and linked within different primary care locations. These electronic data are stored separately from secondary care data, but summaries of individual datasets can be transferred by GPs to secondary healthcare settings. Secondary care data, which are obtained from specialist centres, including hospitals, have, historically, been kept as paper notes, many of which have not been transferred to electronic health record (EHR) systems, such as EPIC and CERNER [4]. Secondary care data are specialist, diverse, complex and in the context of life-long (chronic) diseases, especially, may consist of large longitudinal datasets that require some level of expertise to interpret.

Secondary care data are particularly relevant for patients with IRDs, with other genetic diseases or with chronic disorders, in order to manage their conditions and provide opportunities for therapeutic intervention. Types of secondary care data can include: X-rays, magnetic resonance, coherence tomography or ultrasound images; blood tests; genetic screening or detailed information about surgery. These data, when linked to patients across different hospital visits, are invaluable to medical specialists and, with advances in Artificial Intelligence (AI) and Clinical Decision Support Systems, also have the potential to be of value to non-specialists. Public datasets containing ophthalmological imaging currently exist and have been previously used in machine learning (ML) research but, to date, the reporting of metadata has been poor, rendering them of limited value in research [5].

Genomic data are often stored within the secondary care system in specialised file formats and, as such, are difficult to access and link with other data. Access to genomic data is important as it allows researchers to assess whether genetic variants have been “seen before” and whether they have been previously linked with a disease. It also, crucially, allows design and development of therapies targeted to specific faulty genes. Currently, access to genomic data is only available via the NHS Health and Social Care Network [6], which contain subsets of the data. As datasets expand, there is a growing need for more efficient and secure methods of transferring genomic data between hospitals.

In the UK, NHS staff can gain access to patients’ Summary Care Records, Electronic Prescription Service and e-referrals, stored by NHS Digital, via a service known as the NHS Spine [7]. However, the NHS Spine does not contain any medical imaging data, medical reports or case notes, so this data has limited utility for ongoing medical care. Similar challenges (and potential benefits) exist outside the UK but, to date, there is a global lack of IRD data regarding prevalence, the impact on affected individuals and families, and the cost burden to economies [8].

**References**

[1] Dimitropoulos, L., Patel, V., Scheffler, S. A., Posnack, S., Public attitudes toward health information exchange: perceived benefits and concerns. *The American journal of managed care* 2011, *17*, Sp111-116.

[2] Combs, R., Hall, G., Payne, K., Lowndes, J., Devery, S., Downes, S. M., Moore, A. T., Ramsden, S., Black, G. C., McAllister, M., Understanding the expectations of patients with inherited retinal dystrophies. *Br J Ophthalmol* 2013, *97*, 1057-1061.

[3] Clarke, E., Combs, R., Black, G., Hall, G., Patient expectations and attitudes towards specialist genetic eye services. *J Genet Couns* 2015, *24*, 349-357.

[4] Ratwani, R. M., Savage, E., Will, A., Arnold, R., Khairat, S., Miller, K., Fairbanks, R. J., Hodgkins, M., Hettinger, A. Z., A usability and safety analysis of electronic health records: a multi-center study. *J Am Med Inform Assoc* 2018, *25*, 1197-1201.

[5] Khan, S. M., Liu, X., Nath, S., Korot, E., Faes, L., Wagner, S. K., Keane, P. A., Sebire, N. J., Burton, M. J., Denniston, A. K., A global review of publicly available datasets for ophthalmological imaging: barriers to access, usability, and generalisability. *The Lancet Digital Health* 2021, *3*, e51-e66.

[6] NHS Digital (NHS Health and Social Care Network)

[7] NHS Digital (NHS Spine)

[8] Galvin, O., Chi, G., Brady, L., Hippert, C., Del Valle Rubido, M., Daly, A., Michaelides, M., The Impact of Inherited Retinal Diseases in the Republic of Ireland (ROI) and the United Kingdom (UK) from a Cost-of-Illness Perspective. *Clinical ophthalmology* 2020, *14*, 707-719.

This is a Multimedia Appendix to a full manuscript published in the J Med Internet Res. For full copyright and citation information see http://dx.doi.org/10.2196/jmir.xxxx
