# Supplementary material for: Collaborative Research and Development of a Novel, Patient-Centered Digital Platform (MyEyeSite) for Rare Inherited Retinal Disease Data: Acceptability and Feasibility Study
Source: JMIR Form Res. 2022 Jan 31;6(1):e21341. doi: 10.2196/21341 (PMC8845013; doi:10.2196/21341)
Supplement: Multimedia Appendix 3 [file formative_v6i1e21341_app3.docx]

“MyEyeSite" - Tech/App User Survey (Google Forms)

* Required response

Do you have an inherited retinal eye disease?

- Yes
- No
- Not sure

If yes to above, please indicate your diagnosis:

- Stargardts disease
- Retinitis pigmentosa
- Leber congenital amaurosis
- Other

If 'other', please state your inherited retinal disease diagnosis here:

Do you have any other chronic eye disease (s), which affect your vision?

- Yes
- No
- Not sure

What are your other eye diagnoses (if you have any)?

- Age-related macular degeneration
- Glaucoma
- Cataract
- Diabetic eye disease
- Retinal detachment
- Uveitis or other eye inflammation
- Dry eyes or blepharitis
- Other

Would you like to have a more active role in managing your eye problem?

- Yes
- No
- Not sure

How would you rate your level of sight impairment?

Excellent vision (no sight impairment)

- 1
- 2
- 3
- 4
- 5

Poor vision (severely sight impaired)

Are you able to access to the internet at home or elsewhere (via a desktop computer, laptop or Smartphone)?

- Yes
- No

Do you own a Smartphone ( a mobile phone that connects with the internet)?

- Yes
- No, but I am interested in getting one.
- No, I am NOT interested in Smartphones.
- No, I used to have a Smartphone, but I don't anymore.

If you have a Smartphone, do you download and use apps?

- Yes
- No
- Not applicable

Have you ever made a "subject access request" for your NHS hospital data? For example, have you previously requested copies of your hospital notes, for your own use or to pass onto a third party.

- Yes
- No
- I don't understand what a "subject access request" is

If yes to above, how easy did you find the subject access request process?

Very easy

- 1
- 2
- 3
- 4
- 5

Extremely difficult

Why did you make the subject access request?

Did the subject access request for your data meet the purpose you intended?

- Yes
- No
- Partly

Would you be interested in a personalised app which would allow you to easily store your own hospital data, and share it with people of your choosing?

- Yes
- No
- Maybe

If you answered yes to above, who would manage/ use the app (please indicate all options that apply)?

- Me
- My partner or spouse
- My son or daughter
- Another family member
- A friend
- A professional carer
- My doctor
- Somebody else (not listed above)

What kind of eye data would you be interested in storing in your personal app or web account (please indicate all options that apply) ?

- Medical/ eye diagnoses
- Clinical eye images e.g. retinal photos or OCT scans
- Genetic information/ data relating to eye disease
- Blood test results
- Timeline of my hospital visits and outcomes
- Medications/ prescriptions
- Medical management plans
- Doctors I have seen
- Other data (not listed above)

Who might you choose to share your hospital eye data with (please indicate all options that apply)?

- My consultant ophthalmologist (eye doctor)
- Another consultant ophthalmologist e.g. for a second opinion
- Another hospital specialist (not an ophthalmologist)
- My GP
- My optometrist or optician
- My nurse
- My family and/or friends
- A private healthcare company
- Anybody who might be interested in it
- Other

Do you have any concerns about storing your data in an app or web platform, or concerns about sharing your healthcare/ eye data?

- Yes
- No

If yes, to above, what are your concerns (please indicate all that apply)?

- Whether the data would be useful to me
- Problems that I might have in accessing or using the app/ webpage
- The time it would take to upload/download data to the app/ website
- Data security/ unauthorised access of my data by third parties
- Third party sharing of my data for unethical purposes
- Other

Please describe any further concerns:

Are you:

- Female
- Male
- Non-binary

Ethnic origin

- White
- Asian/ Asian British
- Black/ African/ Caribbean/ Black British
- Mixed/ Multiple Ethnic
- Other

What is your age? *

- Under 12 years old
- 12-17 years old
- 18-24 years old
- 25-34 years old
- 35-44 years old
- 45-54 years old
- 55-64 years old
- 65-74 years old
- 75 years or older

What is the highest level of education that you have completed?

- Primary school
- Secondary school
- Further education/ non-university college qualification
- Trade/ technical/ vocational qualification
- Undergraduate university degree
- Postgraduate university degree
