# Supplementary figures and images for: Collaborative Research and Development of a Novel, Patient-Centered Digital Platform (MyEyeSite) for Rare Inherited Retinal Disease Data: Acceptability and Feasibility Study
Source: JMIR Form Res. 2022 Jan 31;6(1):e21341. doi: 10.2196/21341 (PMC8845013; doi:10.2196/21341)

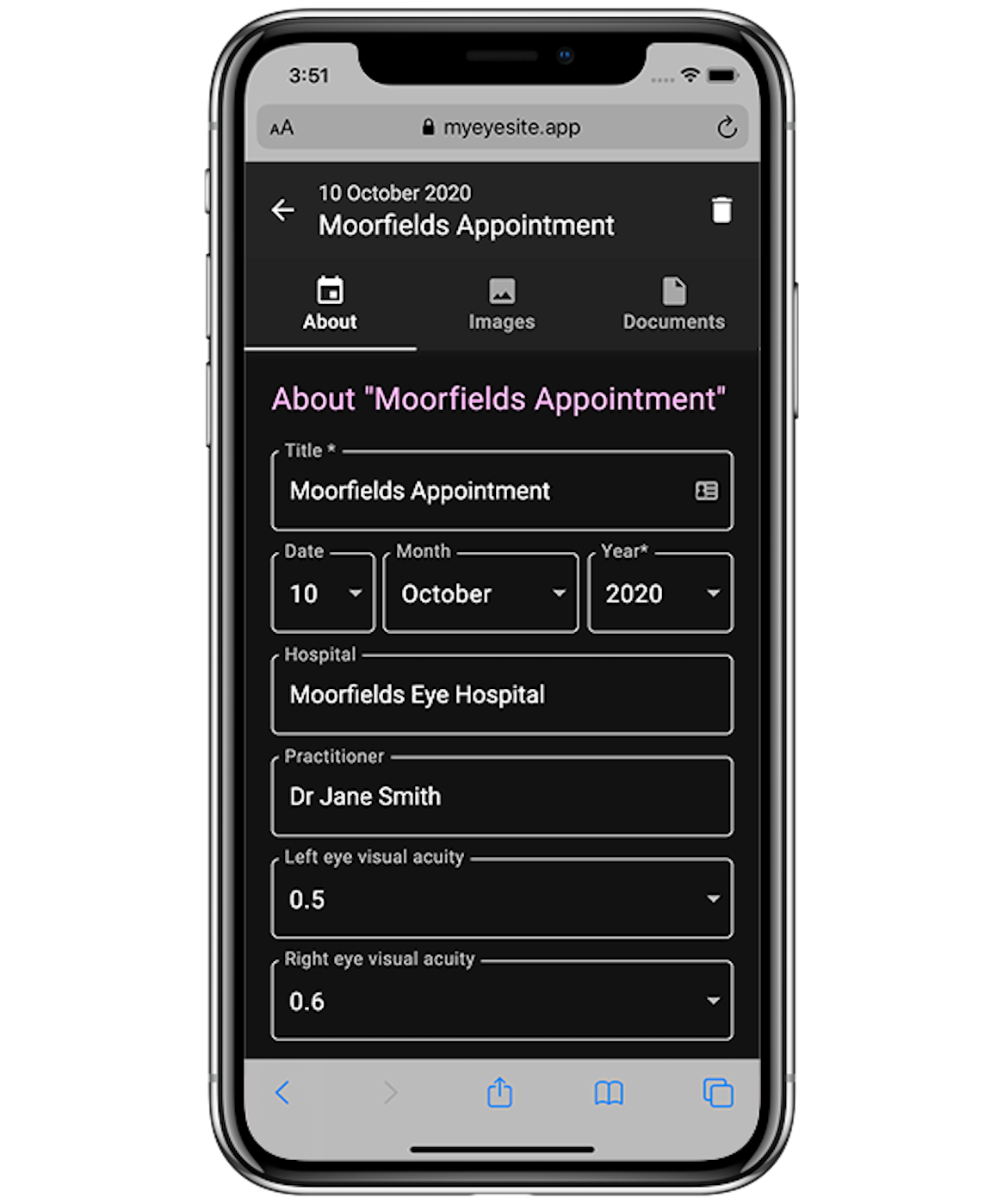

Supplement: Multimedia Appendix 4 [file formative_v6i1e21341_app4.png]

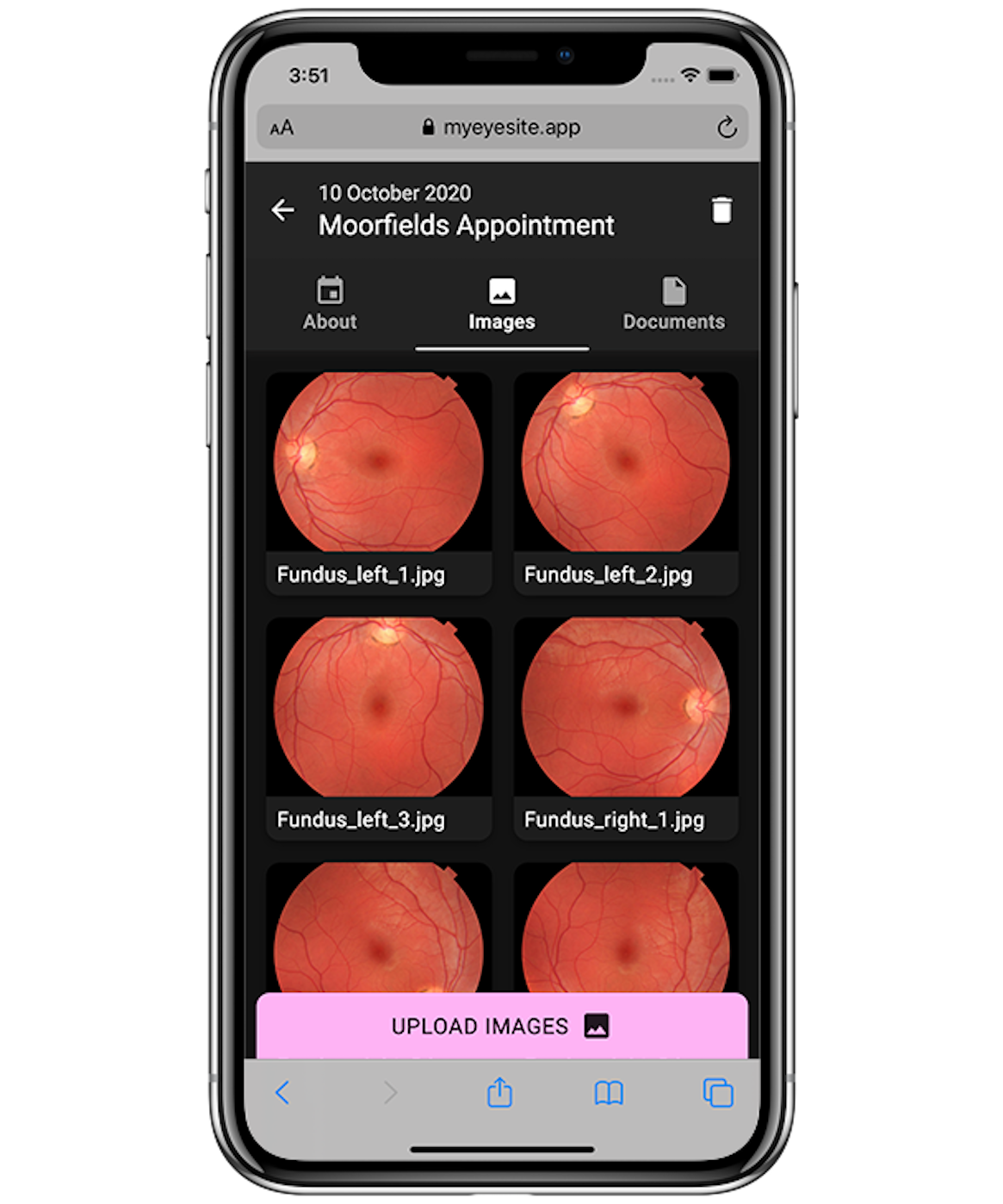

Supplement: Multimedia Appendix 5 [file formative_v6i1e21341_app5.png]
